# Predictors of Rational Management of Diarrhea in an Endemic Setting: Observation from India

**DOI:** 10.1371/journal.pone.0123479

**Published:** 2015-04-07

**Authors:** Tanmay Mahapatra, Sanchita Mahapatra, Barnali Banerjee, Umakanta Mahapatra, Sandip Samanta, Debottam Pal, Nandini Datta Chakraborty, Byomkesh Manna, Dipika Sur, Suman Kanungo

**Affiliations:** 1 Department of Epidemiology, National Institute of Cholera and Enteric Diseases, P-33, CIT Road, Scheme—XM, Beliaghata, Kolkata-700 010, West Bengal, India; 2 Department of Data Management, National Institute of Cholera and Enteric Diseases, P-33, CIT Road, Scheme—XM, Beliaghata, Kolkata-700 010, West Bengal, India; 3 Department of General Medicine, Midnapore Medical College & Hospital, Vidyasagar Rd, Medinipur, West Bengal, 721101‎, India; 4 Department of Pediatrics, Dr. B. C. Roy Memorial Hospital For Children, 111, Narkeldanga Main Road, Phool Bagan, Kolkata—700005, West Bengal‎, India; 5 PATH India Office, A-9 Qutab Institutional Area, USO Road, New Delhi, 110067, India; Beijing Institute of Microbiology and Epidemiology, CHINA

## Abstract

**Background:**

Decades after the establishment of clear guidelines for management, mostly due to irrational approach, diarrhea is still a major concern in the developing world, including India. The scenario is even worse in urban slums owing to poor health-seeking and socio-environmental vulnerability. Determining the distribution of rational diarrhea management by practitioners and identification of its important predictors seemed urgent to minimize the potential for antibiotic resistance, diarrhea-related mortality and morbidity in these areas.

**Methods:**

Between May 2011 and January 2012, 264 consenting, randomly selected qualified and non-qualified practitioners (including pharmacists) were interviewed in the slums of Kolkata, a populous city in eastern India, regarding their characteristics, diarrhea-related knowledge (overall and in six separate domains: signs/symptoms, occurrence/spread, management, prevention/control, cholera and ORS), prescribed antibiotics, intravenous fluid (IVF) and laboratory investigations. Rationality was established based on standard textbooks.

**Results:**

Among participants, 53.03% had no medical qualifications, 6.06% were attached to Governmental hospitals, 19.32% had best knowledge regarding diarrhea. While treating diarrhea, 7.20%, 17.80% and 20.08% respectively advised antibiotics, IVF and laboratory tests rationally. Logistic regression revealed that qualified and Governmental-sector practitioners managed diarrhea more rationally. Having best diarrhea-related knowledge regarding signs/symptoms (OR=5.49, p value=0.020), occurrence/spread (OR=3.26, p value=0.035) and overall (OR=6.82, p value=0.006) were associated with rational antibiotic prescription. Rational IVF administration was associated with best knowledge regarding diarrheal signs/symptoms (OR=3.00, p value=0.017), occurrence/spread (OR=3.57, p value=0.004), prevention/control (OR=4.89, p value=0.037), ORS (OR=2.55, p value=0.029) and overall (OR=4.57, p value<0.001). Best overall (OR=2.68, p value=0.020) and cholera-related knowledge (OR=2.34, p value=0.019) were associated with rational laboratory testing strategy.

**Conclusion:**

Diarrheal management practices were unsatisfactory in urban slums where practitioners’ knowledge was a strong predictor for rational management. Interventions targeting non-qualified, independent practitioners to improve their diarrhea-related knowledge seemed to be required urgently to ensure efficient management of diarrhea in these endemic settings.

## Introduction

Antibiotics are the most frequently used drugs having an upward global trend of usage and the scenario is no different in the developing world including India [1–5]. During 2000–2010, there was 36% escalation in antibiotic use worldwide while Brazil, Russia, India, China and South Africa were responsible for 76% of this increase [2]. Surveys in India revealed that 75–80% of all prescriptions contained at least one antibiotic [1, 6, 7]. Approximately 50% of these antibiotic usages were unfortunately unwarranted as reported by WHO [8].

Diarrhea remained one of the commonest causes of morbidity and mortality, especially among under-five children in the developing world. As most of the diarrheal cases are viral and self-limiting, it is well established for decades that mainstay of management is based on oral rehydration solutions (ORS) and rational use of antibiotics is justified only in a small proportion. Unrestricted use of antibiotics in diarrhea by healthcare providers is still rampant worldwide, more so in the poor-resource settings. In 2010, WHO estimated that less than 60% children with acute diarrhea in developing countries received ORS whereas more than 40% received antibiotic [9]. Evidences of inappropriate antibiotic use in diarrhea are also rising across the globe [1, 9]. A survey in Mexico demonstrated that 37% diarrheal cases received antibiotics although it was indicated only in 5% cases [10]. Report from Indonesia showed that for diarrhea only 46% of under-five children and 36% of those aged more than 5 years received ORS while 73% and 91% of these patients received antibiotics respectively [8]. A survey among Peruvian children found that in 71% common illnesses, antibiotics were prescribed unnecessarily [11]. Overuse of antibiotics for treating childhood diarrhea was observed in a healthcare facility survey in Pakistan [12]. Analysis of prescriptions from public and private medical sectors along with pharmacies in India previously demonstrated that ORS was ignored by more than 90% physicians while treating diarrhea and none of them received any formal training on rational use of drug [5].

According to the WHO factsheet 2010 and other contemporary literatures, inadequate knowledge of healthcare providers, absence of standardized protocol for treatment, lack of proper control on over-the-counter availability of drugs and unethical promotion of new drugs by pharmaceuticals often resulted in improper self-medication, unnecessary use of antibiotics and improper dosing [1, 9, 13, 14]. This misuse has the potential of developing antibiotic resistance among the organisms leading to treatment failures, unwanted adverse effects, enhanced treatment cost to patients as well as huge financial burden on the nation [13, 14]. Anti-microbial resistance has become one of the most serious public health concerns and is currently increasing globally at an alarming rate requiring prompt responses from health sector as well as policy makers [9, 13–15]. According to the 2014 WHO report regarding antibiotic resistance among bacteria causing diarrhea, resistance to fluoroquinolones was very common among *E*. *coli* followed by non-typhoidal *Salmonella* and *Shigella* [16]. The situation seemed to be especially worse in India, where approximately 95% adults carry bacteria resistant to β-lactam antibiotics [17] and strains resistant to newer antibiotics (like fluoroquinolones) were also alarmingly high [18]. Prior evidences also revealed a very high (60–80%) burden of multi-drug resistance among organisms like *E*. *coli*, *Klebsiella spp* in this country [19, 20] along with high level of resistance against azithromycin among pediatric cases of *Campylobacter jejuni* [21].

Due to several infrastructural and logistic shortcomings, management of diarrhea in developing countries like India, till date mainly depends on history, clinical examination and physician’s acumen. Limited research has ever been conducted in India on rationality of diarrheal management by healthcare providers. The situation seemed to be worse among vulnerable populations like urban slums where lack of social security, poor hygiene, very high population density and diverse health-seeking behavior (only 47.6% diarrheas being treated by qualified practitioners in urban slums of Kolkata) of the residents seemed to have the potential for further complicating the scenario [22]. Accurate information on prescription habits of the practitioners in these areas regarding rationality of diarrheal management and their predictors seemed to be the need of the hour to design efficient and target-specific intervention programs to ensure rational diarrheal management.

## Methodology

### Ethics statement

Prior to the interview, details of the study were explained to the practitioners in a language that they understand completely and voluntary written informed consents were obtained from each and every subject maintaining confidentiality as per the standard national guidelines. Data were securely preserved with confidentiality. The study content and procedures were approved (No. C-48/2011-T & E) by the Scientific Advisory Committee and Institutional Ethics Committee of National Institute of Cholera and Enteric Diseases, Kolkata.

### Design

A cross-sectional study [23] was conducted between May 2011 and January 2012, involving a random sample of all allopathic practitioners treating diarrhea in urban slums of eight randomly selected (from altogether 141) administrative units (municipal wards) of Kolkata, India to determine the distribution of rational management of diarrhea and to identify its predictors.

### Eligibility criteria

1. Adults prescribing allopathic medicines to diarrhea patients in the selected wards (28, 29, 30, 32, 33, 34, 59 and 66) for at least last six months were eligible.
2. Did not have any physical/mental condition that prevented proper communications
3. Provided written voluntary informed consent in favor of participation

### Sample size and recruitment

Initially from the list of 141 administrative wards of Kolkata, 8 wards (28, 29, 30, 32, 33, 34, 59 and 66) were randomly selected. Then with administrative and community support, by obtaining lists of practitioners from community based organizations of practitioners like local non-qualified practitioners’ association and the local branch of Indian Medical Association and conducting physical visits by trained community health workers to each clinic (Governmental and private), health centers and pharmacy of the study area in addition, an exhaustive list of 360 eligible qualified and nonqualified practitioners including pharmacists who were prescribing allopathic medicines to diarrhea patients for at least 6 months in the 8 selected wards, was prepared and a unique identification number (UID) was assigned to each of them. Detailed sample size calculation was mentioned elsewhere [23]. Briefly 266 practitioners were required to be interviewed for the estimation of diarrhea-related knowledge and practice of the practitioners using information (variance for knowledge) from the pilot phase and previously observed proportion (0.275) of rational antibiotic use by physicians of Kolkata metropolitan area [24] as the parameter estimate for the sample size calculation (using Epi-info software version-7) assuming α = 0.05 and 10% desired precision [25, 26]. From the list of 360 eligible subjects, based on the UID, 266 were selected randomly using the random number generation method of SAS version-9.2 and invited to participate in the study. Two practitioners refused to participate and thus 264 eligible subjects were recruited for the study after obtaining written informed consent [23].

### Interview

The piloting and of the questionnaire to check internal consistency was mentioned elsewhere [23]. Briefly: 40 practitioners were initially selected randomly from the list of 360 and were subjected to a detailed questionnaire including questions to evaluate their knowledge and practice regarding diarrhea. Using an empirical cut-off value of 0.7 for the deduced Cronbach’s alpha from the collected information in the pilot phase, internally inconsistent questions were removed and the questionnaire was finalized. In the next phase based on this interviewer administered structured questionnaire, face-to-face interview with each participant was conducted as per their convenience regarding venue and timing. Information was collected on the practitioners’ general demographics, category (non-qualified/general/specialist), duration of practice (<10yrs/≥10yrs), attachment (none/private sector/governmental sectors), knowledge regarding diarrhea (overall and in six separate domains: signs/symptoms, occurrence/spread, management, prevention/control, cholera and ORS), most commonly used intravenous fluids (IVF) to correct severe dehydration among diarrhea cases, most commonly advised laboratory test and testing strategy (before/after initiating antibiotics) to identify the causative organism of diarrhea and most commonly prescribed antibiotic for acute watery/bloody diarrhea, mucoid diarrhea and any diarrhea.

### Measures

To estimate knowledge, for each domain, response to individual questions were scored (incorrect = 0 and correct = 1), summed up and rescaled within 10. To measure the overall knowledge, domain-specific scores were added and rescaled within 100. All these domain-specific and overall knowledge scores were then categorized into worst/better/best using tertiles.

Rationality of antibiotic use for different and all types of diarrhea was determined based on the antibiotic treatment guidelines from standard textbooks and observed antibiotic susceptibility patterns among causative organisms of diarrhea in the study area [27–33]. Irrational antibiotic use was defined as use of those antibiotic which were not indicated (because of poor efficacy, commoner side-effect/resistance etc., e.g.: ampicilline in case of acute watery diarrhea) for specific types of diarrhea. Similarly rationality of IVF therapy and laboratory testing advice and strategy were established respectively based on whether ringer lactate/normal saline (rational) or any other fluid (5% dextrose, dextrose-normal saline etc.: irrational) was used to correct severe dehydration among diarrhea cases, whether stool/rectal swab culture was used as the diagnostic test (rational) or not (irrational) and additionally whether testing was advised before antibiotic administration (rational) or not (irrational) [31–34].

### Statistical Analysis

Distribution (frequency, proportion with corresponding standard errors) of the physicians’ characteristics, diarrhea-related knowledge and rationality of management were determined. Logistic regressions were conducted to measure the strength and direction of associations [Odds Ratios (OR), corresponding 95% confidence intervals (95%CI) and p values] between physicians’ characteristics/knowledge and rationality of diarrheal management. SAS version 9.2 was used for statistical analyses.

## Results

Socio-demographic distribution and diarrhea-related knowledge (domain-wise and overall) of the 264 participating practitioners were presented elsewhere [23]. Briefly: most of them (92.05%) were male, 53.03% had no recognized medical qualifications, 25.76% were medical graduates involved in general practice and 21.21% were specialists with postgraduate qualifications. Majority (72.35%) were practicing for more than 10 years, 18.56% were not attached to any healthcare institution, while only 6.06% were working in Governmental hospitals. Different practitioners preferred different IVF for managing severely dehydrated diarrhea cases while only 17.80% and 18.18% respectively used ringer lactate or normal saline principally. Among the participants, 32.95% advised laboratory test of stool to identify the causative organism of diarrhea after initiating antimicrobial therapy and only 26.14% mentioned that for diagnosing the causative organism of diarrhea they advised stool/rectal swab culture. Proportions of participants having best knowledge about diarrheal disease (signs/symptoms), its occurrence/spread, management, prevention/control, cholera, and ORS were as low as: 15.53%, 25.76%, 16.29%, 44.70%, 7.58% and 14.39% respectively while only 19.32% practitioners had best overall knowledge regarding diarrhea. Only 20.08% were rational regarding overall laboratory testing strategy (advice and timing together). While treating cases of acute watery/bloody diarrhea, only 17.05% prescribed antibiotics rationally, for mucoid diarrhea this proportion was 24.62% and overall involving all types of diarrhea it was only 7.20%. Rational use of IVF was observed among 17.80%, 26.14% rationally advised laboratory investigations for diarrhea cases while rational laboratory testing strategy was reported by 20.08% subjects. (Table 1)

**Table 1:** Distribution of the characteristics and diarrheal management practices among participating physicians (N = 264^a^).

Characteristics & management practices	Categories	n ^b^	%	SE ^c^
Category of the practitioners	Non-qualified	140	53.03	3.08
	General	68	25.76	2.70
	Specialist	56	21.21	2.52
Duration of practice	< 10 years	73	27.65	2.76
	≥10 years	191	72.35	2.76
Attached to which type of healthcare facility?	None	49	18.56	2.40
	Private sector	199	75.38	2.66
	Governmental sector	16	6.06	1.47
Intravenous fluid prescribed usually to a case of diarrhea with severe dehydration	5% Dextrose	25	9.47	1.81
	DNS	57	21.59	2.54
	Ringer lactate	47	17.80	2.36
	Normal saline	48	18.18	2.38
	Isolyte-M	7	2.65	0.99
	Others	80	30.30	2.83
Do you ask your diarrhea patients to get laboratory test of stool done before starting antibiotic?	No	87	32.95	2.90
	Yes	177	67.05	2.90
Which laboratory test you advise to diagnose the cause of diarrhea	Blood culture	1	0.38	0.38
	Stool/rectal swab culture	69	26.14	2.71
	Stool for routine microscopy	162	61.36	3.00
	Others	32	12.12	2.01
Rationality of the commonly prescribed antibiotic for the treatment of acute watery or bloody diarrhea	Irrational	219	82.95	2.32
	Rational	45	17.05	2.32
Rationality of the commonly prescribed antibiotic for the treatment of mucoid diarrhea	Irrational	199	75.38	2.66
	Rational	65	24.62	2.66
Rationality of the commonly prescribed antibiotic for the treatment of diarrhea overall	Irrational	245	92.80	1.59
	Rational	19	7.20	1.59
Rationality of the commonly administered IV fluid for the correction of severe dehydration during diarrhea	Irrational	217	82.20	2.36
	Rational	47	17.80	2.36
Rationality of the commonly advised laboratory test type for diagnosis of causative organism for diarrhea	Irrational	195	73.86	2.71
	Rational	69	26.14	2.71
Rationality of the advised laboratory testing strategy for the diagnosis of causative organism for diarrhea	Irrational	211	79.92	2.47
	Rational	53	20.08	2.47

^a^ N = Total number of participating physicians.

^b^ n = Number of participating physicians falling into respective category.

^c^ SE = Standard error.

Logistic regression analyses revealed that compared to the non-qualified practitioners, qualified general practitioners had higher odds of prescribing antibiotics rationally to patients suffering from watery/bloody diarrhea (OR = 3.01, p = 0.007) as well as any type of diarrhea (OR = 5.24, p = 0.019). Specialists with postgraduate qualifications did also show much higher (reference = non-qualified) odds of rationality regarding antibiotic use (for watery/bloody diarrhea: OR = 3.91,p = 0.001; for mucoid diarrhea: OR = 2.22,p = 0.023; for diarrhea overall: OR = 8.75,p = 0.002). Higher duration of practice was associated with increased likelihood of rational antibiotic use in case of watery/bloody diarrhea (OR = 2.87,p = 0.023). Practitioners working in the Governmental sector had considerably higher odds of rational antibiotic use (for watery/bloody diarrhea: OR = 5.11,p = 0.030; for diarrhea overall: OR = 11.08,p = 0.044) compared to those who were not attached to any healthcare institute. The odds of rational antibiotic use by practitioners working in private sector also seemed to be higher than independent practitioners but the analyses lacked power (Table 2).

**Table 2:** Association of physician’s characteristics and knowledge regarding diarrhea with rationality of antibiotic use for diarrheal management (N^a^ = 264).

Practitioners' characteristics and knowledge regarding diarrhea (domain-wise and as a whole)		Categories	Rationality of commonly prescribed antibiotic for treating acute watery/bloody diarrhea		Rationality of commonly prescribed antibiotic for treating mucoid diarrhea		Rationality of Commonly prescribed antibiotic for treating diarrhea overall	
			OR^b^ (95% CI^c^)	p values	OR^b^ (95% CI^c^)	p values	OR^b^ (95% CI^c^)	p values
Category of the practitioner		General	**3.01(1.35–6.67)**	**0.007**	1.33(0.67–2.65)	0.412	**5.24(1.31–20.95)**	**0.019**
(Reference = Non-qualified)		Specialist	**3.91(1.73–8.82)**	**0.001**	**2.22(1.11–4.41)**	**0.023**	**8.75(2.27–33.66)**	**0.002**
Duration of practice (Reference: <10yrs)		≥10yrs	**2.87(1.16–7.09)**	**0.023**	1.23(0.65–2.34)	0.529	2.13(0.60–7.55)	**0.240**
Attached to which type of healthcare facility? (Reference = None)		Private sector	2.48(0.84–7.35)	**0.100**	1.01(0.49–2.08)	0.985	3.91(0.50–30.37)	**0.192**
		Govt. sector	**5.11(1.18–22.26)**	**0.030**	1.03(0.28–3.79)	0.967	**11.08(1.06–115.53)**	**0.044**
Knowledge of the participating physicians regarding	Signs & symptoms of diarrheal diseases (Reference = Worst)	Better	1.43(0.68–3.00)	**0.349**	0.83(0.44–1.56)	0.561	2.81(0.75–10.49)	**0.125**
		Best	2.13(0.85–5.36)	**0.107**	1.80(0.82–3.95)	0.141	**5.49(1.30–23.13)**	**0.020**
	Occurrence and spread of diarrhea (Reference = Worst)	Better	0.91(0.41–1.99)	**0.808**	0.97(0.49–1.93)	0.936	0.27(0.05–1.42)	**0.122**
		Best	1.48(0.65–3.38)	**0.351**	1.67(0.81–3.47)	0.166	**3.26(1.09–9.77)**	**0.035**
	Management of diarrhea (Reference = Worst)	Better	**3.07(1.43–6.59)**	**0.004**	1.51(0.81–2.82)	0.199	2.29(0.76–6.94)	**0.142**
		Best	2.16(0.81–5.80)	**0.126**	1.73(0.78–3.84)	0.175	2.26(0.58–8.83)	**0.243**
	Prevention and control of diarrhea (Reference = Worst)	Better	2.04(0.81–5.15)	**0.133**	1.33(0.59–2.99)	0.493	3.78(0.81–17.58)	**0.090**
		Best	1.03(0.40–2.68)	**0.950**	1.30(0.60–2.85)	0.509	1.13 (0.21–6.01)	**0.888**
	Cholera as a whole (Reference = Worst)	Better	**3.18(1.52–6.62)**	**0.002**	1.49(0.83–2.67)	0.185	2.77(0.93–8.22)	**0.067**
		Best	**4.29(1.39–13.21)**	**0.011**	1.24(0.41–3.70)	0.702	4.48(0.98–20.46)	**0.053**
	Oral rehydration solution and its use (Reference = Worst)	Better	1.26(0.62–2.58)	**0.523**	**1.92(1.03–3.56)**	**0.039**	2.30(0.75–7.10)	**0.147**
		Best	1.83(0.74–4.49)	**0.189**	1.79(0.78–4.12)	0.172	3.61(0.99–13.21)	**0.053**
	Diarrhea as a whole (Reference = Worst)	Better	**2.93(1.35–6.34)**	**0.006**	1.90(0.99–3.63)	0.052	3.19(0.82–12.37)	**0.094**
		Best	2.26(0.89–5.73)	**0.085**	2.13(0.99–4.56)	0.053	**6.82(1.73–26.92)**	**0.006**

Boldfaced figures denote results for which P<0.05.

^a^ N = Total number of participating physicians.

^b^ OR = Odds ratio.

^c^ 95%CI = 95% Confidence Interval.

In comparison with those having worst knowledge in the respective domains, practitioners having better knowledge regarding management of diarrhea (OR = 3.07,p = 0.004), better (OR = 3.18,p = 0.002) and best (OR = 4.29,p = 0.011) knowledge regarding cholera had higher odds of rational antibiotic use in cases of acute watery/bloody diarrhea. Subjects with better knowledge (reference = worst) regarding ORS (OR = 1.92,p = 0.039) were also more likely to prescribe rational antibiotics while treating mucoid diarrhea cases. Having best knowledge (reference = worst) regarding signs/symptoms of diarrheal diseases (OR = 5.49,p = 0.020) and occurrence/spread of diarrhea (OR = 3.26,p = 0.035) were associated with higher likelihood of rational antibiotic prescription in any type of diarrhea. (Table 2)

Compared to the non-qualified practitioners, qualified general practitioners had higher odds of administering IVF rationally (OR = 3.75, p<0.001), advising rational laboratory tests (OR = 3.30, p<0.001) for diarrhea and following rational laboratory testing strategy (OR = 4.36, p<0.001). Postgraduate specialists also had much higher (reference = non-qualified) odds of rationality for administered IVF (OR = 2.72, p = 0.018) and laboratory investigations (for advice: OR = 3.95, p<0.001; for strategy: OR = 5.02, p<0.001). Physicians from Governmental sector had considerably higher odds of rational IVF administration (OR = 6.97, p = 0.016) and laboratory investigation (for advice: OR = 6.59,p = 0.003; for strategy: OR = 11.25,p<0.001) compared to those who were not attached to any healthcare institute. The odds of rational IVF administration (OR = 3.74, p = 0.034) by practitioners working in private sector also seemed to be higher than independent practitioners. Rational administration of IVF for correction of severe dehydration was more likely by the practitioners having best knowledge (reference = worst) regarding diarrheal signs/symptoms (OR = 3.00, p = 0.017), occurrence/spread (OR = 3.57,p = 0.004), prevention/control (OR = 4.89,p = 0.037) and ORS (OR = 2.55,p = 0.029). Better knowledge (reference = worst) regarding management (for better knowledge: OR = 2.00,p = 0.028) and prevention/control (for best knowledge: OR = 2.67,p = 0.023) were associated with higher odds of rationality in laboratory testing advice while better knowledge (reference = worst) about cholera (for best knowledge: OR = 2.34,p = 0.019) and management of diarrhea (for better knowledge: OR = 2.28,p = 0.021) were associated with increased likelihood of rational laboratory testing strategy. (Table 3)

**Table 3:** Association of physician’s characteristics and knowledge regarding diarrhea with rationality of fluid management and laboratory testing practices while treating diarrhea cases (N^a^ = 264).

Practitioners' characteristics and knowledge regarding diarrhea (domain-wise and as a whole)		Categories	Rationality of commonly administered IV fluid for the correction of severe dehydration		Rationality of commonly advised laboratory test for diagnosis of causative organism		Rationality of laboratory testing strategy for the diagnosis of causative organism	
			OR^b^(95% CI^c^)	p values	OR^b^ (95% CI^c^)	p values	OR^b^ (95% CI^c^)	p values
Category of the practitioner		General	**3.75(1.75–8.02)**	**<0.001**	**3.30(1.67–6.48)**	**<0.001**	**4.36(2.02–9.41)**	**<0.001**
(Reference = Non-qualified)		Specialist	**2.72(1.19–6.24)**	**0.018**	**3.95(1.95–8.00)**	**<0.001**	**5.02(2.27–11.11)**	**<0.001**
Duration of practice (Reference: <10yrs)		≥10yrs	0.79(0.39–1.54)	**0.472**	1.52(0.80–2.92)	**0.204**	1.59(0.77–3.29)	**0.212**
Attached to which type of healthcare facility? (Reference = None)		Private sector	**3.74(1.10–12.65)**	**0.034**	1.81(0.80–4.12)	**0.156**	2.92(0.99–8.58)	**0.052**
		Govt. sector	**6.97(1.44–33.68)**	**0.016**	**6.59(1.90–22.88)**	**0.003**	**11.25(2.73–46.35)**	**<0.001**
Knowledge of the participating physicians regarding	Signs & symptoms of diarrheal diseases (Reference = Worst)	Better	1.65(0.78–3.51)	**0.193**	1.04(0.56–1.92)	**0.896**	1.56(0.78–3.13)	**0.211**
		Best	**3.00(1.22–7.41)**	**0.017**	1.62(0.73–3.58)	**0.232**	2.05(0.85–4.96)	**0.110**
	Occurrence and spread of diarrhea (Reference = Worst)	Better	1.39(0.59–3.29)	**0.457**	1.24(0.63–2.41)	**0.536**	1.36(0.63–2.94)	**0.433**
		Best	**3.57(1.51–8.47)**	**0.004**	1.56(0.75–3.26)	**0.233**	2.23(0.99–5.01)	**0.052**
	Management of diarrhea (Reference = Worst)	Better	1.53(0.75–3.12)	**0.243**	**2.00(1.08–3.70)**	**0.028**	**2.28(1.14–4.57)**	**0.021**
		Best	1.88(0.78–4.53)	**0.163**	1.83(0.82–4.08)	**0.138**	2.29(0.96–5.49)	**0.063**
	Prevention and control of diarrhea (Reference = Worst)	Better	**9.89(2.24–43.62)**	**0.003**	1.85(0.76–4.49)	**0.175**	1.47(0.57–3.81)	**0.428**
		Best	**4.89(1.10–21.83)**	**0.037**	**2.67(1.15–6.22)**	**0.023**	2.14(0.87–5.26)	**0.097**
	Cholera as a whole (Reference = Worst)	Better	1.51(0.70–3.23)	**0.295**	0.88(0.44–1.78)	**0.722**	0.89(0.40–1.98)	**0.773**
		Best	1.83(0.84–3.97)	**0.127**	1.80(0.92–3.50)	**0.086**	**2.34(1.15–4.75)**	**0.019**
	Oral rehydration solution and its use (Reference = Worst)	Better	1.03(0.50–2.12)	**0.940**	0.79(0.43–1.47)	**0.458**	0.94(0.48–1.86)	**0.861**
		Best	**2.55(1.10–5.91)**	**0.029**	1.88(0.87–4.03)	**0.107**	2.03(0.89–4.60)	**0.091**
	Diarrhea as a whole (Reference = Worst)	Better	1.36(0.61–3.01)	**0.452**	**1.97(1.04–3.72)**	**0.037**	**2.36(1.15–4.84)**	**0.020**
		Best	**4.57(2.03–10.27)**	**<0.001**	**2.19(1.03–4.64)**	**0.041**	**2.68(1.17–6.15)**	**0.020**

^a^ N = Total number of participating physicians.

^b^ OR = Odds ratio.

^c^ 95%CI = 95% Confidence Interval.

Practitioners having better (reference = worst) overall knowledge regarding diarrhea, had higher odds of rationality while prescribing antibiotics to patients suffering from acute watery/bloody diarrhea (for best knowledge: OR = 4.57,p<0.001), mucoid diarrhea (for better: OR = 1.97,p = 0.037 & best knowledge: OR = 2.19,p = 0.041) and any type of diarrhea (for better: OR = 2.36,p = 0.020 & best knowledge: OR = 2.68,p = 0.020), while administering IVF (for better knowledge: OR = 2.93,p = 0.006) and in the laboratory testing strategy (for best knowledge: OR = 6.82,p = 0.006). (Tables 2 & 3)

## Discussion

Involving a representative sample of 264 practitioners prescribing allopathic medicines to diarrhea patients for at least six months, this study revealed that less than half (46.97%) of the practitioners treating diarrheal diseases in urban slums of Kolkata, a highly populous Metro city in eastern India, were qualified. This finding corroborated with prior observations in similar setting in India and other countries in the developing world, regarding childhood as well as adult diarrhea. [22, 35–40].

Among all the participants, majority was practicing for long (≥10 years) and very few (6.06%) were attached to Governmental facilities. Similar observation was reported from other studies conducted in Pakistan and Peru where proportion of diarrhea cases presented to public sector was very low and very few of the practicing physicians belonged to public sector [12, 41–43].

Knowledge of the physicians regarding different domains of diarrheal diseases and management was far below satisfactory level, while less than 20% (19.32%) of the practitioners had best overall knowledge regarding diarrhea. This unfortunately low knowledge regarding diarrhea had also been reported by others [40, 43, 44]. An investigation among practitioners from Iraq and Afghanistan previously showed that less than one-third practitioners could correctly identify common causes of diarrhea while only 30% had correct knowledge about management [44]. Another survey in seven Latin American and Caribbean countries reported that physicians had inadequate knowledge regarding diarrhea and its antibiotic management [15].

In the current study only 17.05% practitioners were found to prescribe antibiotics rationally while treating acute watery/bloody diarrhea. Prior research did also show that in case of bloody diarrhea irrationality of antibiotic use was very likely (OR = 19.04) [10]. The scenario was shed better in case of mucoid diarrhea (24.62%) while for all types of diarrhea the overall situation was very poor (only 7.20% prescribed antibiotics rationally). Inappropriate and overuse of antibiotics for diarrheal treatment were found to be rampant across the globe and urgent intervention to prevent this misuse seemed to be the need of the hour [6, 10, 39, 45–47].

Regarding fluid management of diarrhea, 64.02% practitioners were prescribing irrational IVFs to correct severe dehydration. The observed proportion was comparable to several previous studies in similar poor-resource setting and much higher than other developed areas [32, 40, 43, 44].

While advising laboratory investigations to identify causative organism of diarrhea, 73.86% were irrational regarding the suggested test and 79.92% practitioners mentioned irrational testing strategy overall. Irrational laboratory investigation for diarrheal patients were also observed by others and training programs to improve specific practices should be implemented urgently [43, 48].

Qualified general practitioners and specialists were much more likely to advise antibiotics, IVF and laboratory investigations rationally compared to their non-qualified counterparts. Intuitively enough this observation supported previous findings and established the need for urgent interventions to bring non-qualified practitioners under the coverage of regular training schedule and monitoring to improve overall management of diarrhea in settings where non-qualified practitioners would remain an integral part of healthcare [40, 42, 49].

Longer duration of practice was associated with higher odds of rational antibiotic use in case of watery/bloody diarrhea. An exploratory research involving physicians in peri-urban area of Lima, Peru previously illustrated that length of practice was an important predictor of appropriate prescription habit among practitioners [53] while some other studies also reported similar influence of practitioners’ experience on management practices [3, 40, 50].

Practitioners attached to Government hospitals had much higher likelihood of rational antibiotic use, fluid management and laboratory investigations while treating diarrhea cases compared to those who were only doing independent practices and for practitioners working in private sector the scenario in terms of rationality also seemed to be better than those practitioners without attachments. These findings corroborated with previous studies where public sector physicians were found to be more likely to prescribe antibiotics rationally than others [6, 12, 40, 42, 43].

Practitioners’ domain-wise and overall knowledge regarding diarrheal diseases, their prevention/control and management seemed to be major drivers of their rational diarrhea management practices. Better knowledge about diarrheal management and cholera was significantly associated with rational antibiotic use in acute watery/bloody diarrhea. Knowledge regarding ORS was positively correlated with the likelihood of better antibiotic treatment of mucoid diarrhea. Those having best knowledge about signs, symptoms, occurrence and spread of diarrhea had higher odds of prescribing antibiotics rationally in any type of diarrheas compared to their less knowledgeable counterparts. Although similar observation was reported from several studies in comparable settings [15, 40, 44, 51–53], lack of association between knowledge and rationality were also observed among practitioners regarding antibiotic use [38, 43, 47]. Factors like patient/caregivers’ preferences, affordability and severity of diarrhea were other strong predictors [38, 40, 46, 47, 50, 54–56].

Rational fluid therapy was significantly predicted by best knowledge regarding diarrheal signs/symptoms, occurrence/spread, prevention/control and ORS. Physicians’ knowledge and other patient related factors were found to have strong correlations in other studies also [3, 32, 57].

Corroborating with prior explorations, it was found in our study that, relatively improved knowledge about diarrheal management, prevention/control and cholera was associated with higher odds of rational laboratory testing advice and strategy. [52, 58]

Practitioners having better overall knowledge regarding diarrhea were much more likely to prescribe rational antibiotics, administer appropriate IVF to correct severe dehydration and advise laboratory investigations rationally while managing diarrhea cases. Improvement of overall diarrheal management was also evidenced with betterment of relevant knowledge in most of the prior studies conducted among practitioners [40, 43, 51, 52].

Current study had some important limitations. Due to the cross-sectional design, causal interpretation of the observed associations may not be recommended and any effort to extrapolate the results beyond the study population should be made with caution. Self-reported nature of the data and questionnaire-based evaluation of knowledge/rationality of practice could have introduced some potential for information bias. To minimize the potential for non-compliance, the questionnaire had to be relatively less time consuming for the busy practitioners. Hence detailed information on all potential confounders could not be collected. Our study area only had 360 practitioners eligible to be recruited. Among these practitioners it was not possible to conduct a study with sufficient power for the multivariate analyses. Also due to budgetary constraints, it was not possible for us logistically to enlarge the study area. Hence we had to be content with the bivariate analyses that we conducted. Thus possibility of residual confounding remained.

Despite these limitations, by virtue of representative sampling, excellent participation and detailed algorithm-based measurements it was concluded that the current study could provide important insight into the role of knowledge in rational management of diarrheal diseases among vulnerable slum-dwellers of Kolkata by practitioners. Multi-component educational interventions to improve the knowledge of the practitioners regarding diarrheal diseases, their management and prevention seemed to be required urgently, specifically targeting the non-qualified, independent practitioners including pharmacists to ensure efficient management and control of diarrheal diseases in this area.
